# Routine use of positron-emission tomography/computed tomography for staging of primary colorectal cancer: Does it affect clinical management?

**DOI:** 10.1186/1477-7819-11-49

**Published:** 2013-02-27

**Authors:** Gokhan Cipe, Nurhan Ergul, Mustafa Hasbahceci, Deniz Firat, Suleyman Bozkurt, Naim Memmi, Oguzhan Karatepe, Mahmut Muslumanoglu

**Affiliations:** 1 Faculty of Medicine, Department of General Surgery, Bezmialem Vakif University, Adnan Menderes Bulvari, Istanbul, Fatih 34090, Turkey; 2 Faculty of Medicine, Department of Nuclear Medicine, Bezmialem Vakif University, Adnan Menderes Bulvari, Istanbul, Fatih 34090, Turkey

**Keywords:** Colorectal cancer, Computed tomography, Positron emission tomography/computed tomography, Preoperative staging

## Abstract

**Background:**

The use of positron emission tomography-computed tomography (PET/CT) for the preoperative staging of patients with colon and rectal cancer has increased steadily over the last decade. The aim of this study was to evaluate the effect of PET/CT on the preoperative staging and clinical management of patients with colorectal cancer.

**Methods:**

Between December 2010 and February 2012, 64 consecutive patients with colorectal cancer were evaluated with both PET/CT scans and conventional preoperative imaging studies. We prospectively recorded the medical reports of these patients. The PET/CT findings were compared with conventional imaging studies and the rate of over-staging or down-staging and changes in clinical management were evaluated. The correlation of the PET/CT with the conventional imaging was compared by a kappa agreement coefficient. Differences in the accuracy for N and T staging were assessed by *χ*2 and related-samples marginal homogeneity tests.

**Results:**

Thirty-nine (60.9%) patients had rectal cancer and 25 (39.1%) had colon cancer. Based on PET/CT, additional lesions were found in 6 (9.4%) of the patients: hilar and paratracheal lesions in 4 patients, hepatic in 1 and supraclavicular in 1 patient. In four of six patients, detailed imaging studies or biopsies revealed chronic inflammatory changes. Hepatic and supraclavicular involvement was confirmed in two patients. Therefore, the false positivity rate of PET/CT was 6.25%. Based on the additional PET/CT, 2 (3.2%) patients had a change in surgical management. A chemotherapy regimen was administered to the patient with a 1.5 cm hepatic metastasis near the right hepatic vein; for another patient with an identified supraclavicular lymph node metastasis, a simultaneous excision was performed.

**Conclusions:**

Routine use of PET/CT for preoperative staging did not impact disease management for 96.8% of our patients. The results of our study conclude that PET/CT should not be routinely used for primary staging of colorectal cancer. More studies are required for identifying the subgroup of patients who might benefit from a PET/CT in their initial staging.

## Background

Colorectal malignancies are the third most common cancer and a common cause of cancer related death [1]. Accurate preoperative staging of colorectal carcinoma (CRC) is essential for evaluating the expected prognosis and providing the optimal treatment strategy [2]. The depth of invasion, occurrence of lymph node metastases and distant metastases are the main factors that affect the prognosis of the patient [3]. Preoperative assessment and staging of colorectal cancer is often problematic. A colonoscopy is a well-known procedure for inspection of the colon, but this procedure does not provide sufficient evidence about the depth of the tumor, lymph node involvement and distant metastases [4]. Although technical refinements have provided better quality for conventional imaging (CI) studies such as multi detector computed tomography (MDCT) and magnetic resonance imaging (MRI), complete correct preoperative staging has not yet been obtained. A recent study showed that the overall accuracy of MDCT was 86% in T staging and 84% in N staging [5]; another study reported that MRI provided an 81% overall agreement with histological findings for the T and N stages [6].

The use of 18 F-fluorodeoxyglucose-positron emission tomography/computed tomography (PET/CT) has allowed for better staging and resulted in clinical management variations when applied to a number of tumors [7,8]. Currently, PET/CT is recommended only for the assessment of the suspected recurrence of CRC and in pre-operative staging prior to metastasectomy. Furthermore, the clinical opinion on the role of PET/CT in the routine management of primary colon cancer varies. Some investigators suggest that in certain clinical circumstances like the initial staging of primary rectal cancer, this method is going to be considered as part of the standard preoperative assessment in the near future [9].

The aim of this study was to prospectively evaluate the effect of PET/CT on patients with primary colorectal cancer, including an assessment of the stage and of clinical management planning.

## Methods

This study had been approved by the Bezmialem Vakif University Ethical Committee (Number: B.30.2.BAV.0.05.05/262), and we obtained written informed consent from all patients included in the study.

Between December 2010 and February 2012, consecutive patients with currently diagnosed colorectal cancer in our tertiary hospital were included in the study. In addition to CI studies, PET/CT was performed on these consecutive patients, and all data were prospectively entered into a database. All patients’ diagnoses were confirmed by histo-pathology before they were enrolled in the study. If the patients were classified as having Stage II or III rectal carcinoma, they had been referred for neo-adjuvant therapy. All PET/CT scans were performed before preoperative chemo-radiotherapy.

Patients were excluded if preoperative CI studies or PET/CT was not performed at our institution, if the patients had a recurrent disease or if the patients were unwilling to participate in the study.

The CI modalities included an examination with abdomino-pelvic MDCT and MRI.

All preoperative MDCT studies were performed using an MDCT scanner with 64 parallel detector rows (Toshiba Aquilion 64, Toshiba Medical Systems Corp., Tokyo, Japan). Nonionic intravenous contrast material (Omnipaque 300, Amersham Health, Princeton NJ, USA) was administered at a dose of 2 mL/kg up to a maximum of 180 mL.

MRI was performed with a 1.5 T MRI system (MagnetomAvanto, Siemens Healthcare, Erlangen, Germany) with a phased-array coil. Either gadodiamide (Omniscan, Amersham Health) or gadobutrol (Gadovist, Schering) was used as the contrast agent.

PET/CT scans were acquired on a Siemens Biograph 16 PET/CT System (Siemens medical solutions, Knoxville, TN) at least one hour after intravenous injection of 18 F-fluorodeoxyglucose (FDG). All patients fasted for six hours prior to the study, but were encouraged to drink water. Patients also received bowel preparation prior to the procedure. All PET/CT images were interpreted by one nuclear medicine specialist prior to surgery. The mean interval between the CI studies and PET/CT was 6 days (range 1–12 days).

T and N staging was based on the international TNM classification, as follows: pT1, tumor invading submucosal layer; pT2, tumor invading muscularis propria or subserosa; pT3, tumor penetrating serosa and perivisceral fat; and pT4, tumor invading adjacent organs. Lymph nodes were likewise classified: N0, no regional lymph node metastasis; N1, metastasis in one to three perirectal lymph nodes; N2, metastasis in four or more perirectal lymph nodes; and N3, metastasis in pelvic lymph nodes.

The PET/CT scan analysis for liver metastases was based on apparently visualized activity greater than normal liver parenchyma. Abnormalities where the FDG uptake was less than the physiological liver uptake were not considered to be positive. For chest activity, the focal uptake of FDG needed to be greater than the mediastinal uptake and needed to correspond to an anatomic structure or abnormality seen by the CI studies.

A single radiologist with 5 years of experience in abdomino-pelvic MDCT and MRI evaluated the images. All patients with CRC were staged according to the tumor/node/metastasis (TNM) classification described by the American Joint Committee on Cancer (AJCC) for CRC [10]. The PET/CT findings were directly correlated with previous CI images and the rate of over-staging or down-staging and change in management were evaluated.

Results of the CI studies were regarded as the standard reference point for PET/CT. Sensitivity, specificity, positive predictive value, negative predictive value and accuracy of PET/CT were calculated for T and N staging considering the results of CI studies. The correlation of the PET/CT with the CI studies was compared by a kappa agreement coefficient. Differences in the accuracy for N and T staging were assessed by *χ*2 and related-samples marginal homogeneity tests. SPSS 19.0 software (Chicago, Illinois, USA) was used for statistical analysis. The level of statistical significance with 0.95 confidence limits was set at *P* = 0.05.

## Results

Sixty-four consecutive patients underwent PET/CT scanning. The mean age was 59 years (range 18–85 years), and 69% were male. Thirty-nine patients had rectal cancer and underwent MRI, while 25 patients had colon cancer and underwent a MDCT scan. Results of CI studies with regard to T and N features of the patients were summarized in Table 1. It was possible to detect all primary lesions during CI studies.

**Table 1:** T and N staging of all colorectal cancers

		**T staging**				**N staging**			
**Localization**	**Method**	**T1**	**T2**	**T3**	**T4**	**N0**	**N1**	**N2**	**N3**
Rectum (*n* = 39)	PET/CT	-	8	26	3	19	16	4	-
	CI studies	-	11	23	5	16	19	3	1
Colon (*n* = 25)	PET/CT	1	6	16	2	20	5	-	-
	CI studies	-	6	14	5	6	18	1	-

The accuracy, sensitivity, specificity, positive predictive value and negative predictive value for PET/CT assessment based on the results of CI studies were calculated separately for the T and N stages . The sensitivity and accuracy of PET/CT for T staging was also higher than for N staging (Table 2).

**Table 2:** Evaluation of the efficacy of PET/CT for T and N staging

	**Sensitivity (%)**	**Specificity (%)**	**Positive predictive value (%)**	**Negative predictive value (%)**	**Accuracy (%)**
T staging	95.74	75	91.83	85.71	90.47
N staging	52.38	85	88	47.36	63.49

The κ agreement coefficient analysis showed that the correlation between PET/CT and CI studies were higher with the T staging than the N staging. The kappa value for the T staging was found to be 0.604 and for N staging was 0.283. These results were statistically significant (Table 3).

**Table 3:** Correlation of PET/CT with CI studies

	**Kappa value**	**p value**
T staging	0.604	<0.001
N staging	0.283	0.002

After comparison PET/CT and CI findings for the same patients with regard to T and N stages, statistically significant differences between PET/CT and CI studies in the T and N stages of the same patients were found (for T stages: *χ*^2^ = 29.93, *P *= 0.025; for N stages: *χ*^2^ = 42.84, *P* = 0.0001) (Table 3).

PET/CT accurately identified the primary tumor in all patients. Comparing PET/CT with CI studies, there were incidental findings in 6 (9.4%) patients. According to the PET/CT results, the preoperative stage of 14 patients (21.9%) changed. Down staging was found in 8 patients, while over staging was found in six patients (Table 4).

**Table 4:** Changing pattern in patients’ stage as determined by PET/CT

	**Change of stage**	**Patients (%)**
Down-staging	Stage-III → Stage-II	3 (4.7)
	Stage-II → Stage-I	5 (7.8)
Over-staging	Stage-II → Stage-IV	2 (3.1)
	Stage-III → Stage-IV	4 (6.2)
Total		14 (21.8)

PET/CT examination revealed suspected findings for metastasis in mediastinum in 4 patients, liver metastasis in 1 patient, and left supraclavicular metastasis in 1 patient. However, 4 of these patients were found to be mistakenly over-staged in which the situation later was confirmed by biopsies or further imaging studies. All of the mediastinal metastases were false positive (Table 5). Furthermore, this process caused a delay in the surgical treatment for these patients.

**Table 5:** Additional distant lesions detected by PET/CT

**Localization**	**N**	**Verification**
Mediastinal (hilar, paratracheal)	4 (false positive)	Histology
Liver	1	Not confirmed histologically
Left supraclavicular lymph node	1	Excision and pathological examination

**Figure 1:**
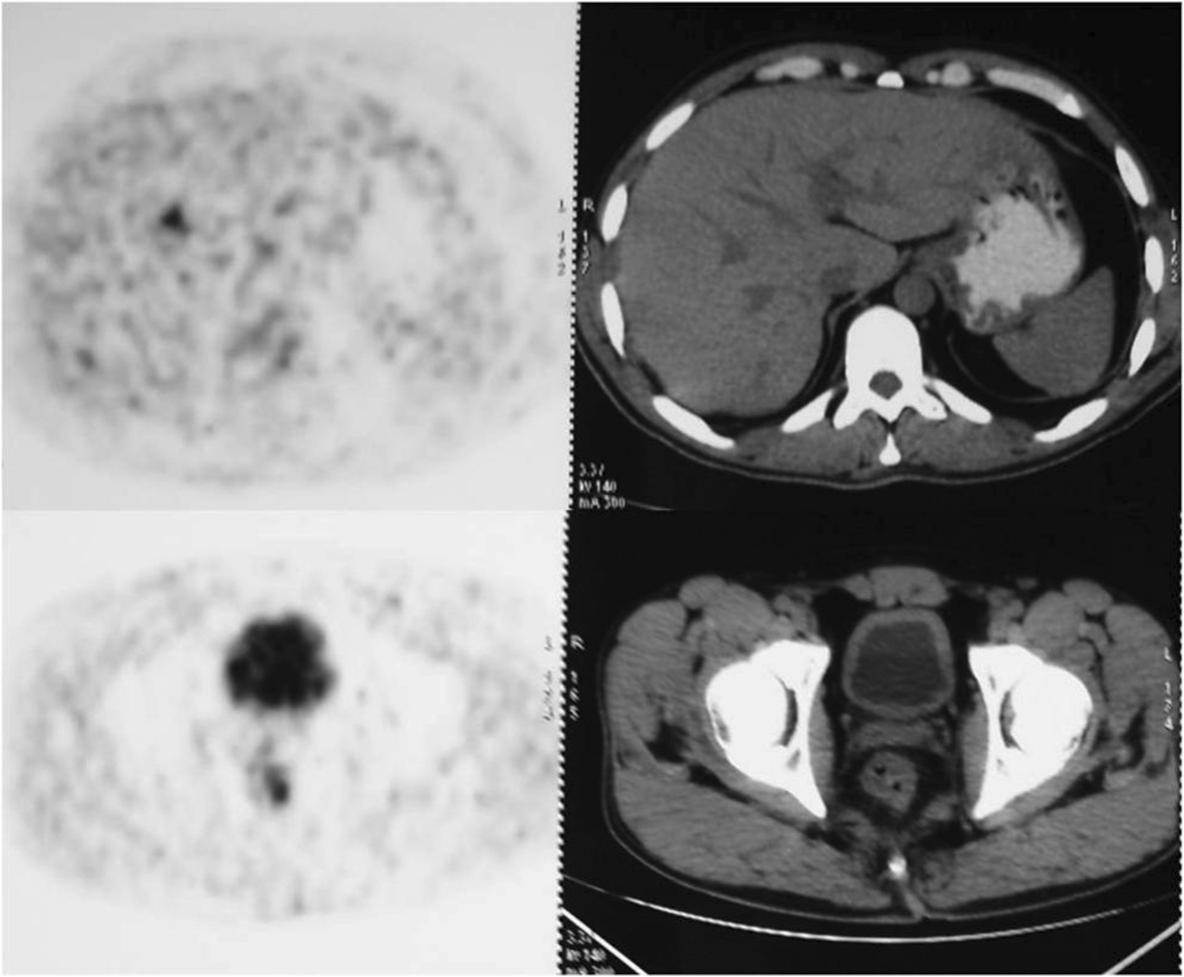
Appearance of primary tumor and hepatic metastases in PET/CT image.

**Figure 2:**
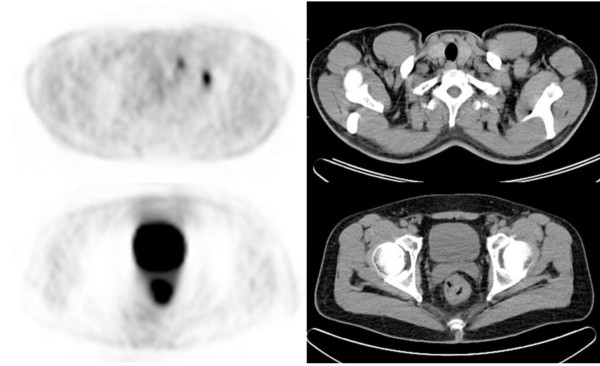
Appearance of primary tumor and left supraclavicular lymph node metastases in PET/CT image.

Additional findings from the PET/CT changed the treatment strategies for two patients (3.2%). PET/CT identified a liver metastasis in one patient who could not be shown by CI studies (Figure 1). A chemotherapy regimen for metastatic disease was administered to the patient for a 1.5 cm hepatic metastasis near the right hepatic vein. In the second patient, isolated supraclavicular lymph node metastasis was detected by PET/CT (Figure 2), which changed the surgical treatment strategy for this patient. A simultaneous supraclavicular lymph node excision and total mesorectal excision were performed in this patient.

## Discussion

Surgery is the main treatment with curative potential for recurrent and metastatic (mainly liver) colorectal cancer. The presence of the disease at a site distant to the planned surgery affects the type and timing of treatments. Together, this wide variation in disease presentations and extents of treatment underpins the rationale for accurate pre-operative staging. Although colonoscopy is the most common method for detecting and diagnosing colorectal cancers, it does not produce accurate preoperative information regarding tumor invasion and lymph node involvement. For this purpose, MDCT and MRI are used as the standard modality for preoperative staging of colorectal cancers. Moreover, neoadjuvant therapies are performed for stage II and stage III rectal cancer. It has been shown that neoadjuvant therapy decreases local recurrence and increases survival [11]. Therefore, correct preoperative staging has a critical role in determining whether patients should undergo neoadjuvant therapy. Similarly, preoperative neoadjuvant strategies for colonic cancers are dependent on the staging accuracy of CT [12]. Unfortunately, CT cannot provide complete correct staging of colorectal cancer, even though an improvement in the resolution has recently been achieved with MDCT.

Use of MDCT on determination of T and N staging has variable results [5,13-16]. Although it has been reported up to 86% of accuracy for T staging, this rate has been decreased to 59% for N staging. Lack attenuation differences between tumor and normal visceral softy tissue, and inadequate distension of the bowel are thought to be responsible for low sensitivity and specificity of MDCT for T staging [17]. MDCT can be considered to be more efficacious for N staging than T staging. However, efficacy of MDCT on determination of lymph node status of CRC has not been showed by many studies. Due to heterogeneity on the design of the studies and technical differences, it has been impossible to get generally accepted results.

Likewise, MRI offers an 81% overall accuracy compared with histological findings for T and N staging [6]. Due to these imperfect results of CI studies, authors have investigated new preoperative imaging modalities for colorectal cancer; some authors suggest the use of PET/CT as an alternative option [15,18,19]. It has been shown that the accuracy of PET/CT can be as high as 94.3% for T staging. However, sensitivity and specificity for N staging still remains to be low in comparison to T staging [20]. Positive lymph nodes that are smaller than 1 cm may be the major source for being missed by PET/CT [18]. In this study, it has been shown that PET/CT has the accuracy rate of 90.47% for T staging. Although it can be regarded as an acceptable rate for T staging, lack of both pathological confirmation and comparison with CI studies attenuate the reliability of our results.

In addition to provide accurate staging, the ability of PET/CT to detect metastatic disease is thought to be a critical point for its potential therapeutic impact. However, studies have reported inconsistent findings about the effect of PET/CT on clinical practice and surgical management. Some studies found no effect and others reported decreased morbidity from improved surgical techniques arising from increased precision in tissue identification. In some studies, PET/CT was compared directly with CT alone, which is one example of a falsely enhanced apparent therapeutic impact for PET/CT. PET/CT has commonly been performed for the detection of recurrence or for routine follow-up in patients with colorectal cancer. The sensitivity and specificity of PET/CT in patients with recurrent colorectal cancer was found to be 97% and 76%, respectively [14]. However, PET imaging provides insufficient anatomical information; this lack of information was improved with the integration of CT into PET imaging. Cohade et al. showed that the accuracy of PET alone and PET/CT in preoperative staging of colorectal cancer was 78% and 89%, respectively [14]. MRI also provides additional accuracy to liver contrast-enhanced CT in the assessment of a patient’s suitability for hepatic resection.

There are limited studies in the literature that have investigated PET/CT in the preoperative staging of colorectal cancer. By using PET/CT as a preoperative imaging modality, it was reported that the stages of the tumors were changed in 27% to 39% of the patients either down- or over-stages [21-24]. Our data showed that the preoperative stage changed in 21.9% of the cases according to the PET/CT results; this result is comparable to findings in the literature. However, a modification of clinical management including only the surgical treatment modality was found in only 3.2% of the cases. In eight and six cases, there were down and over staging of the primary tumor which have no effect on the choice of surgical treatment, respectively. Any modifications with regard to postoperative adjuvant treatment caused by preoperative PET/CT were beyond the primary aim of this study, which was to evaluate the effect of PET/CT on surgical treatment. It has been reported that use of PET/CT in staging of rectal cancer resulted in discordant and incidental findings in an almost half of the cases. Although PET/CT brought on stage migration in 30% of the cases, either down or over staging, potential management changes occurred only in 25% and there was no need to change the surgical management [25]. This discordance may be explained by a high false positivity rate of PET/CT for detecting distant metastasis. PET/CT detected 6 distant metastases, which could not be shown by CI studies. Four of the 6 distant metastases were verified as false positive by biopsies (Table 5). The high rate of false positive results which was caused by the high accumulation of FDG in mediastinal lymph nodes and hilar region might be related to the high incidence of chronic infectious and inflammatory diseases of the chest in our country. The present study suggests that some additional evidence offered by PET/CT was not always beneficial and caused preoperative diagnostic dilemmas, which caused further invasive examinations, additional costs and a delay of the disease management. Selecting patients with locally advanced tumors in whom distant metastases are more expected and patients with suspected metastasis detected with other imaging modalities for PET/CT scanning may be more beneficial and practical for clinical use.

Local staging of colorectal cancer mostly depends on CI studies. There are various studies regarding CT and MRI that reported a high success rate. The specificity and sensitivity of CT and MRI for the detection of adjacent organ invasion were reported as comparable [26]. PET/CT is inappropriate to determine the exact depth of invasion of the primary tumor due to its limited resolution. However, PET/CT may be appropriate in selected cases to estimate penetration and local invasion. MDCT provides more accurate anatomical and structural information than PET. Therefore, T staging of colorectal cancer by PET/CT is almost completely reliant on CT. As expected, the present study demonstrated a close correlation of PET/CT with CI studies in T staging (Tables 1 and 3).

The main problem of staging of colorectal cancer is the prediction of lymph node involvement. The sensitivity of CT for the detection of lymph node involvement has been reported to be between 29% and 90% for CRC [13,25]. PET/CT showed low sensitivity (52%) and relatively high specificity (85%) for detecting lymph node involvement in the present study. The overall accuracy of PET/CT (63%) was below the expected value. Several previous studies have reported a comparable rate of lymph node involvement detection by PET/CT, reporting low sensitivity (29–37%) and high specificity (83–96%) [25,27,28].

A full assessment of the colon is mandatory to localize the tumor, to evaluate locoregional spreading, and to depict synchronous colonic lesions. For that purpose, MDCT is the most favored imaging technique. However, CT might have poor performances for determining local tumor extension in the absence of colon distension [29]. CT colonography is another exam which is primarily used to evaluate the colon in cases of incomplete colonoscopy and as an alternative means of screening for colorectal carcinoma [30,31]. Although both contrast enhancement by using intravenous agents to define the boundaries of structures, and colonography to identify primary tumor with its local extent increases the accuracy of such modalities, the choice of PET/CT without intravenous contrast medium or colonography has been shown to be effective, especially in T staging of CRC [18]. Moreover, contrast-enhancement causes more accurate N staging of rectal cancer compared with non-contrast-enhancement during PET/CT examination. PET/CT colonography is also used in preoperative diagnosis of the tumors proximal to obstructive colorectal cancers, which were defined as cancers that cannot be traversed colonoscopically [32]. Use of this technique has been reported to have an overall accuracy of 80% and 60% for the evaluation of tumor depth and lymph nodes, respectively. Use of water enema or air-contrast enema during CT colonography may also result in better evaluation of the local spread for T staging for CRC [31,33]. However, FDG as a radiotracer may play the role of “metabolic contrast agent”. By that way, it can be helpful to increase the contrast resolution of the structures, to characterize the perilesional tissues and to compensate for the absence of luminal distension on the unenhanced CT images [18]. Therefore, identification of the primary tumor with its local extent by using FDG PET/CT, without administration of intravenous contrast medium or colonography can be possible as supported by our results.

The high false-negative rate of PET/CT may be attributable to the limited resolution and proximity of the involved lymph nodes to the locally advanced primary tumor or the urinary bladder. In addition, while a lymph node with a micro-metastasis and a diameter >5 mm can be considered to be involved by CT assessment, the same lymph node can be considered as non-metastatic by PET/CT because no FDG uptake is detected. These results imply that preoperative PET/CT is of limited value for detecting metastasis to regional lymph nodes.

There were four suspected pulmonary and one supraclavicular metastasis in our patients. Although chest CT before PET/CT could help to differentiate the malignant potential of such lesions, use of chest CT in staging of CRC remains controversial [17,34]. It was shown that chest CT altered the initial TNM stage in less than 1% of CRC patients. In addition, indeterminate lung nodules were found to be positive in almost one quarter of the patients [34]. In the light of these findings, chest CT was not used as the primary staging method in this study.

The main limitation of our study was the wide variety of pathological groups and subgroups of the primary tumors that may have influenced the sensitivity and accuracy of PET/CT for TNM staging of colorectal cancer, as FDG uptake may differ among tumor types. Although the contribution of PET/CT to the detection of recurrent and metastatic colorectal cancer has been reported in many current studies, its value in staging the primary disease has not been well-defined and usually is not recommended as a first-line diagnostic tool in clinical practice. Although most of the tumors presented in this study had T staging of T2 and T3, and N staging of N0 and N1, it could be difficult to generalize the results to all subgroups of colorectal cancers. In addition, presence of both colonic and rectal cancers, and application of the neo-adjuvant treatment to Stage II and III rectal cancers might be the other confounding variables to affect the reliability of our results. Therefore, more studies that include special subgroups of colon and rectal cancers are necessary to determine the role of PET/CT in primary staging.

PET/CT seems to be a useful tool in the evaluation of colorectal cancer by allowing to metabolically characterizing undetermined lesions suspected for recurrence of disease, to perform a complete pre-surgical staging and to identify occult metastatic disease. However since it is an expensive modality and the impact to the management of disease may be low as in our study; its use in routine preoperative examination is controversial.

Another aspect to be considered for the routine use of PET/CT as a first-line diagnostic modality is contrast-enhanced PET/CT scanning, which may replace the routine contrast-enhanced CT imaging; this method will allow for whole body detection of distant metastases and show the primary tumor and loco-regional lymph nodes more accurately. Studies with contrast-enhanced PET/CT scans are needed in the future.

## Conclusions

The use of PET/CT for preoperative staging did not impact the clinical management of 96.8% of our patients. The present study revealed that PET/CT does not provide higher diagnostic precision to CI modalities in the detection of primary tumors, lymph node involvement or distant metastases. These results suggest that PET/CT should not be routinely used for primary staging of colorectal cancer. More studies are required to select the patients who might benefit from PET/CT during initial staging.

## Abbreviations

CRC: Colorectal cancer; CI: Conventional imaging; MDCT: Multi-detector computed tomography; MRI: Magnetic resonance imaging; CT: computed tomography; 18F PET/CT: Fluorodeoxyglucose positron emission tomography/computed tomography; FDG: Fluorodeoxyglucose.

## Competing interests

The authors declare that they have no competing interests.

## Authors’ contributions

GC participated in the study concept and design, and carried out data acquisition, analysis, and interpretation of data and drafting of the manuscript; NE participated in the study design and helped to draft the manuscript; MH, DF, SB, and NM participated in the study and helped data acquisition; OK, and MM participated in the study carried out critical revisions. All authors read and approved the final manuscript.

## References

[B1] Boyle P Ferlay J Cancer incidence and mortality in Europe Ann Oncol 2005 16 481 488 10.1093/annonc/mdi098 15718248

[B2] Sun CH Li ZP Meng QF Yu SP Xu DS Assessment of spiral CT pneumocolon in preoperative colorectal carcinoma World J Gastroenterol 2005 11 3866 3870 15991284 10.3748/wjg.v11.i25.3866 PMC4504887

[B3] Flippone A Ambrosini R Fuschi M Marinelli T Genovesi D Bonomo L Preoperative T and N staging of colorectal cancer: accuracy of contrast- enhanced multi-detector row CT colonography-initial experience Radiology 2004 231 83 90 10.1148/radiol.2311021152 14990815

[B4] Miao YM Amin Z Healy J Burn P Murugan N Westaby D Allen-Mersh TG A prospective single centre study comparing computed tomography pneumocolon against colonoscopy in detection of colorectal neoplasms Gut 2000 47 823 837 10.1136/gut.47.6.832 PMC1728155 11076883

[B5] Ahmetoglu A Cansu A Baki D Kul S Cobanoglu U Alhan E Ozdemir F MDCT with multiplanar reconstruction in the preoperative local staging of rectal tumor Abdom Imaging 2011 36 31 37 10.1007/s00261-009-9591-y 19949791

[B6] Giusti S Buccianti P Castagna M Fruzzetti E Fattori S Castelluccio E Caramella D Bartolozzi C Preoperativerectal cancerstaging with phased-array MR Radiat Oncol 2012 7 29 10.1186/1748-717X-7-29 22390136 PMC3310712

[B7] Mac Manus MP Hicks RJ Ball DL Kalff V Matthews JP Salminen E Khaw P Wirth A Rischin D McKenzie A F-18 fluorodeoxyglucose positron emission tomography staging in radical radiotherapy candidates with nonsmall cell lung carcinoma: powerful correlation with survival and high impact on treatment Cancer 2001 92 886 895 10.1002/1097-0142(20010815)92:4<886::AID-CNCR1397>3.0.CO;2-V 11550162

[B8] Leong T Everitt C Yuen K Condron S Hui A Ngan SY Pitman A Lau EW MacManus M A prospective study to evaluate the impact of FDG-PET on CT-based radiotherapy treatment planning for oesophageal cancer Radiother Oncol 2006 78 254 261 10.1016/j.radonc.2006.02.014 16545881

[B9] Kalff V Hicks R Ware R Hogg A Binns D McKenzie A The clinical impact of 18 F-FDG PET in patients with suspected or confirmed recurrence of colorectal cancer: a prospective study J Nucl Med 2002 43 492 499 11937593

[B10] Park JS Choi GS Hasegawa S Sakai Y Huh JW Kim HR Kwak SG Validation of the seventh edition of the American Joint Committee on cancer tumor node-staging system in patients with colorectal carcinoma in comparison with sixth classification J Surg Oncol 2012 106 674 679 10.1002/jso.23117 22514036

[B11] Selçuk D Demirel K Ozer H Baca B Hatemi I Mihmanli I Korman U Oğüt G Comparison of virtual colonoscopy with conventional colonoscopy in detection of colorectal polyps Turk J Gastroenterol 2006 17 288 293 17205408

[B12] Iyer RB Silverman PM DuBrow RA Charnsangavej C Imaging in the diagnosis, staging, and follow-up of colorectal cancer AJR Am J Roentgenol 2002 179 3 13 10.2214/ajr.179.1.1790003 12076894

[B13] Påhlman L Glimelius B Improved survival with preoperative radiotherapy in resectable rectal cancer N Engl J Med 1997 336 980 987 9091798 10.1056/NEJM199704033361402

[B14] Dighe S Blake H Koh MD Swift I Arnaout A Temple L Barbachano Y Brown G Accuracy of multidetector computed tomography in identifying poor prognostic factors in colonic cancer Br J Surg 2010 97 1407 1415 10.1002/bjs.7096 20564305

[B15] Kwak JY Kim JS Kim HJ Ha HK Yu CS Kim JC Diagnostic value of FDG-PET/CT for lymph node metastasis of colorectal cancer World J Surg 2012 36 1898 1905 10.1007/s00268-012-1575-3 22526032

[B16] Duman M Tas S Mecit EA Polat E Duman U Kurtulus BA Varolgunes H Bostanci EB Preoperative local staging of colorectal cancer patients with MDCT Hepatogastroenterology 2012 59 1108 1112 22281979 10.5754/hge11869

[B17] Tan CH Iyer R Use of computed tomography in the management of colorectal cancer World J Radiol 2010 2 151 158 10.4329/wjr.v2.i5.151 21161029 PMC2999018

[B18] Mainenti PP Iodice D Segreto S Storto G Magliulo M De Palma GD Salvatore M Pace L Colorectal cancer and 18FDG-PET/CT: what about adding the T to the N parameter in loco-regional staging? World J Gastroenterol 2011 17 1427 1433 10.3748/wjg.v17.i11.1427 21472100 PMC3070015

[B19] Uchiyama S Haruyama Y Asada T Hotokezaka M Nagamachi S Chijiiwa K Role of the standardized uptake value of 18-fluorodeoxyglucose positron emission tomography-computed tomography in detecting the primary tumor and lymph node metastasis in colorectal cancers Surg Today 2012 42 956 961 10.1007/s00595-012-0225-6 22711186

[B20] Lu YY Chen JH Ding HJ Chien CR Lin WY Kao CH A systematic review and meta-analysis of pretherapeutic lymph node staging of colorectal cancer by 18 F-FDG PET or PET/CT Nucl Med Commun 2012 33 1127 1133 10.1097/MNM.0b013e328357b2d9 23000829

[B21] Akiyoshi T Oya M Fujimoto Y Kuroyanagi H Ueno M Yamaguchi T Koyama M Tanaka H Matsueda K Comparison of preoperative whole-body positron emission tomography with MDCT in patients with primary colorectal cancer Colorectal Dis 2009 11 464 469 10.1111/j.1463-1318.2008.01643.x 18637927

[B22] Huebner RH Park KC Shepherd JE Schwimmer J Czernin J Phelps ME Gambhir SS A meta-analysis of the literature for whole-body FDG PET detection of recurrent colorectal cancer J Nucl Med 2000 41 1177 1189 10914907

[B23] Heriot A Hicks R Drummond E Keck J Mackay J Chen F Kalff V Does positron emission tomography change management in primary rectal cancer? A prospective assessment Dis Colon Rectum 2004 47 451 458 10.1007/s10350-003-0089-3 14978612

[B24] Gearhart S Frassica D Rosen R Choti M Schulick R Wahl R Improved staging with pretreatment positron emission tomography / computed tomography in low rectal cancer Ann Surg Oncol 2006 13 397 404 10.1245/ASO.2006.04.042 16485158

[B25] Eglinton T Luck A Bartholomeusz D Varghese R Lawrence M Positron-emission tomography/computed tomography (PET/CT) in the initial staging of primary rectal cancer Colorectal Dis 2010 12 667 673 19486092 10.1111/j.1463-1318.2009.01873.x

[B26] Davey K Heriot A Mackay J Drummond E Hogg A Ngan S Milner A Hicks R The impact of 18-Flourodeoxyglucose positron emission tomography-computed tomography on the staging and management of primary rectal cancer Dis Colon Rectum 2008 51 997 1003 10.1007/s10350-008-9244-1 18461399

[B27] Bipat S Glas AS Slors FJM Zwinderman AH Bossuyt PMM Stoker J Rectal cancer: local staging and assessment of lymph node involvement with endoluminal US, CT, and MR imaging—a meta-analysis Radiology 2004 232 773 783 10.1148/radiol.2323031368 15273331

[B28] Furukawa H Ikuma H Seki A Yokoe K Yuen S Aramaki T Yamaguchi S Positron emission tomography scanning is not superior to whole body multidetector helical computed tomography in the preoperative staging of colorectal cancer Gut 2006 55 1007 1011 10.1136/gut.2005.076273 16361308 PMC1856325

[B29] Soyer P Hamzi L Sirol M Duchat F Dray X Hristova L Placé V Pocard M Boudiaf M Colon cancer: comprehensive evaluation with 64-section CT colonography using water enema as intraluminal contrast agent-a pictorial review Clin Imaging 2012 36 113 125 10.1016/j.clinimag.2011.06.010 22370132

[B30] Utano K Endo K Togashi K Sasaki J Kawamura HJ Horie H Nakamura Y Konishi F Sugimoto H Preoperative T staging of colorectal cancer by CT colonography Dis Colon Rectum 2008 51 875 881 10.1007/s10350-008-9261-0 18350337

[B31] da Fonte AC Chojniak R de Oliveira FF Pinto PN dos Santos Neto PJ Bitencourt AG Inclusion of computed tomographic colonography on pre-operative CT for patients with colorectal cancer Eur J Radiol 2012 81 e298 e303 10.1016/j.ejrad.2011.10.017 22100372

[B32] Nagata K Ota Y Okawa T Endo S Kudo SE PET/CT colonography for the preoperative evaluation of the colon proximal to the obstructive colorectal cancer Dis Colon Rectum 2008 51 882 890 10.1007/s10350-008-9236-1 18330647

[B33] Nagata K Endo S Kudo SE Kitanosono T Kushihashi T CT air-contrast enema as a preoperative examination for colorectal cancer Dig Surg 2004 21 352 358 10.1159/000081543 15479978

[B34] McQueen AS Scott J CT staging of colorectal cancer: what do you find in the chest? Clin Radiol 2012 67 352 358 10.1016/j.crad.2011.10.005 22169348

